# Evaluation of the genotoxicity of PM2.5 collected by a high-volume air sampler with impactor

**DOI:** 10.1186/s41021-019-0120-0

**Published:** 2019-02-28

**Authors:** Kazutoshi Sugita, Yuka Kin, Mayuko Yagishita, Fumikazu Ikemori, Kimiyo Kumagai, Toshihiko Ohara, Makoto Kinoshita, Kazuyuki Nishimura, Yukihiko Takagi, Daisuke Nakajima

**Affiliations:** 1 Azabu University, 1-17-71, Fuchinobe, Chuou-ku, Sagamihara, Kanagawa 252-5201 Japan; 2 Prefectural University of Hiroshima, Nanatsuka 5562, Syoubara, Hiroshima 727-0023 Japan; 3 Nagoya City Institute for Environmental Sciences, 16-8,Toyoda 5-chome, Minami-ku, Nagoya, 457-0841 Japan; 4 Gunma Prefectural Institute of Public Health and Environmental Sciences, 378 Kamioki-machi, Maebashi, Gunma 371-0052 Japan; 5 Health environment center, Hiroshima Prefectural Technology Research Institute, 1-6-29, Minami-machi, Minami-ku, Hiroshima-shi, Hiroshima, 734-0007 Japan; 6 Fukuoka City Institute for Hygiene and the Environment, 2-1-34, Jigyohama, Chuo-ku, Fukuoka city, 810-0065 Japan; 7 National Institute for Environmental Studies, 16-2 Onogawa, Tsukuba, Ibaraki, 305-8506 Japan

**Keywords:** PM2.5, High-volume air sampler, Impactor, Mutagenic activity, Microsuspension, TA98, TA100, Dibenzo [a,l]pyrene

## Abstract

**Background:**

The harmful effects of fine particles with an aerodynamic diameter less than 2.5 μm (PM2.5) on respiratory organs are emphasized in pollution studies because PM2.5 have high deposition rates in the respiratory organs and contain various hazardous compounds. In this study, a sampling method combining a high-volume air sampler (HV) with a PM2.5 impactor was developed for collecting large quantities of PM2.5. The concentrations of elemental carbon (EC), organic carbon (OC), inorganic ions, and polycyclic aromatic hydrocarbons (PAHs) were measured in PM2.5 collected by the high-and low-volume air samplers (LV).

**Results:**

Similar results were obtained from the HV and LV methods, with respect to inorganic carbon, organic carbon, sodium ions, ammonium ions, and PAHs with more than four rings. Because of the much larger amount of PM2.5 could be collected by the HV method, the trace constituents, that were difficult to detect by the conventional LV method, were readily detected by the HV method. Furthermore, when the microsuspension method that was modified more sensitive Ames mutagenicity test, was used to test the PM2.5 samples at four sites, mutagenic activities were detected by strains TA100 and TA98. Most of the mutagenic activity was associated with the PM2.5 fraction and mutagenic activity in winter was greater than that in summer.

**Conclusions:**

The HV method produced results similar to those from the conventional LV method with respect to the PM2.5 components present in the atmosphere in relatively high concentrations, but its 40-fold greater flow rate enabled the detection of mutagenic compounds present in only trace concentrations.

## Background

Recent reports in Japan have indicated decreases in the emission of suspended particulate matter (SPM) [40], polycyclic aromatic hydrocarbons (PAHs) [7] and dioxins into the atmosphere, by emission control on car engines and improved management of waste incineration. On the other hand, it has been reported that the Japanese atmosphere is influenced by sulfur oxides from coal combustion from China [30], so there is considerable interest in Japan regarding air pollution by PM2.5 from China ([39], Ling et al. 2016). PM2.5 are widely studied because of their harmful influence on human health, such as cardiovascular disease and respiratory disorders, as a result of the high deposition rate in the respiratory organs [28]. Various epidemiological and toxicological studies have shown strong positive correlations between PM2.5 concentrations in the atmosphere and the death rate by respiratory system disease [8], and a direct influence on collective human mortality [4, 26, 27]. Furthermore, PAHs, such as benzo [a] pyrene (BaP, [1, 3, 22, 29]), nitro-PAHs, and dioxins [28], which are related compounds with carcinogenic activity, have been detected in PM2.5. PAHs derived from atmospheric suspended particulates were shown to be retained in the lungs of a dog [32]. The World Health Organization (WHO) has set guidelines for PM2.5 in the atmosphere at 10 μg/m^3^ for the annual average concentration and at 25 μg/m^3^ for the daily average. The continuous monitoring of PM2.5 in Japan is essential, and an environmental standard of PM2.5 in the atmosphere was set in 2009 at 15 μg/m^3^ for the annual average and at 35 μg/m^3^ for the daily average, and continuous monitoring is carried out in all parts of Japan. PM2.5 is collected by LV as part of the monitoring, but chemical analysis of the samples collected is limited because only small samples can be collected by this method, which may be below the detection limit of some pollutants. Therefore, equipment collecting larger amounts of PM2.5 is needed.

It is currently impossible to measure all the potentially carcinogenic substances contained in PM2.5 samples because of the large number of compounds present at very low concentrations. Therefore, to achieve a comprehensive evaluation of the harmful effects of substances associated with PM2.5, a mutagenic assay, such as the Ames test, is needed. On the other hand, the conventional Ames mutagenicity test uses *Salmonella typhimurium* strains TA98 (in which frameshift mutations are detected) and TA100 (in which base substitution mutations are detected) which cannot detect mutagenic activity because the sensitivity is not high enough, and the amount of PM2.5 collected is limited. Therefore, modified Ames tests have been developed in which the mutagenicity of PM2.5 samples has been increased using TA98 and a TA98-derivative strain, YG1024, with elevated *O*-acetyltransferase levels. The YG1024 strain shows hyper-responsiveness to nitro-PAHs and amino-PAHs [6, 36], but there is the risk of introducing a bias toward these compounds when this modified Ames test is used.

In the current study, parallel sampling of PM2.5 was carried out using the HV and LV methods, with concentrations of EC, OC, some ion components, and PAHs being compared in PM2.5 collected by the two sampling methods, to evaluate the validity of collecting PM2.5 using the new HV method. Subsequently, PM2.5 and the particle bigger than PM2.5 (PM > 2.5) were collected using the HV method, and the carcinogenic potential was evaluated using the TA98 and TA100 strains, these being the strains used for the conventional Ames test, to achieve a comprehensive evaluation of the carcinogenesis-related substances in PM2.5 and PM > 2.5 collected by the HV method.

## Methods

### Materials

Acetone (HPLC grade; FUJIFILM Wako Pure Chemical Corporation, Oosaka, Japan) and dimethyl sulfoxide (DMSO, Dojindo Molecular Technologies, Inc., Kumamoto, Japan) were used for extraction. MgSO_4_•(7H_2_O), citric acid hydrate, dihydrogen phosphate ammonium, dihydrogen phosphate potassium, sodium hydroxide, D-glucose (The reagent best quality, FUJIFILM Wako Pure Chemical Corporation, Oosaka, Japan) and agar (Nacalai Tesque Inc., Kyoto, Japan) were used for a mutation assay.

*S. typhimurium* strains TA98 and TA100 were distributed from National Institute of Public Health.

### PM2.5 sampling

The PM2.5 samples were collected at four locations in Japan, namely Maebashi (East longitude 36.4045°, North latitude 139.0961°), Tsukuba (East longitude 36.0498°, North latitude 140.1175°), Nagoya (East longitude 35.0990°, North latitude 136.9156°) and Fukuoka (East longitude 33.5948°, North latitude 130.3646°). At Nagoya, parallel sampling by the LV and HV methods was carried out to measure the concentrations of EC, C and some ion components in the spring and summer 2012 and winter 2013 (21 samples) and to measure the concentrations of PAHs in the summer 2013 and winter 2014 (15 samples). At the other sampling points, sampling by the HV method was carried out to measure the mutagenic activities in the summer 2012 and winter 2013 (64 samples including 14 samples in Nagoya).

**Fig. 1:**
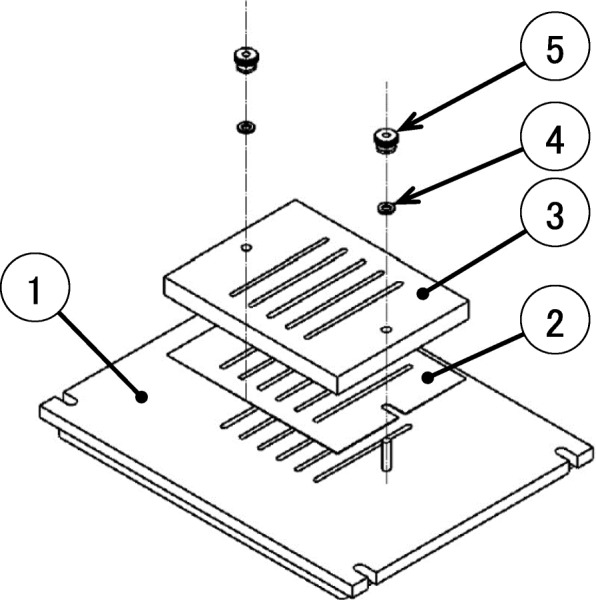
Schematic of PM2.5 impactor for high-volume air sampler (HV-1000 PM 2.5 impactor).① Base of impactor, ②; filter with slits, ③; Nozzle, ④; Washer, ⑤; Nut

Samples of PM2.5 were collected on 47 mmφ quartz-fiber filters (PALLFLEX 2500QAT-UP, PALL corporation, New York, USA) for a 24 h period by the LV method and on 20.3 × 25.4 cm quartz-fiber filters (PALLFLEX 2500QAT-UP, PALL corporation, New York, USA) by the HV method. A low-volume air sampler, Partisol 2000-FRM Air Sampler (Thermo Fisher Scientific, Massachusetts, USA) was used with a flow rate of 16.7 L/min as LV method. In 2000-FRM, PM2.5 were collected by back-up filter, and PM > 2.5 were eliminated by impactor that consist of filter and pump oil [25]. This PM 2.5 collection method is certified by the Federal Reference Law of the United States and is consistent with the manual of the Japanese Ministry of the Environment. A high-volume air sampler, HV-700R (SIBATA Scientific Technology Ltd., Saitama, Japan) was used with a flow rate of 700 L/min as HV method. In the HV method, an impactor for PM2.5 (Custom-made based on a HV-100 2.5 impactor, SIBATA Scientific Technology Ltd., Saitama, Japan) was mounted on HV and PM2.5 was collected on pre-treated back-up quartz-fiber filters, and PM > 2.5 was collected on slit quartz-fiber filters (2500QAT-UP, PALL corporation, New York, USA) referring Fig. 1. Each quartz-fiber filter was heat-treated at 450 °C for 2 h in a muffle furnace to remove organic pollutants and then equilibrated in a desiccator at a constant 50% humidity and weighed.

### Analysis of carbonaceous and ion components

A 1.0 cm^2^ sample of the quartz-fiber filter with aerosol particles, collected using both the LV and HV, was placed in an oven. Then, OC and EC were analyzed with a laboratory OC-EC aerosol analyzer (Sunset Laboratory Inc., Oregon, USA) following the Interagency Monitoring of Protected Visual Environments (IMPROVE) thermal/optical reflectance protocol [2].

The quartz-fiber filter of 4 cm^2^ in HV method and 5 cm^2^ in LV method was subjects to ultrasonic treatment with 10 ml of pure water to extract ion species respectively. After extraction, filter debris and suspended insoluble particles were removed using a Teflon filter (DISMIC-25HP, Advantec Toyo Kaisha, Ltd., Tokyo, Japan). Cation species such as Na^+^, NH_4_^+^, K^+^, Mg^2+^ and Ca^2+^ and anion species such as Cl^−^, NO_3_^−^ and SO_4_^2−^ were measured by ion chromatograph (Dionex ICS-1000, Thermo Fisher Scientific, Massachusetts, USA).

### Analysis of polycyclic aromatic hydrocarbons

One-half of the quartz-fiber filter collected by the LV method and one-fourth of the quartz-fiber filter collected by the HV method were used for PAH measurements. The targeted PAHs (including dibenzo [a,l] pyrene (DBalP)), for gas chromatography-mass spectrometry (GC/MS) analysis are shown in Table 1. A 16 PAHs mixture (representing the U.S. EPA priority PAHs) (each 20 ng) labeled with deuterium was added to the filter sample as an internal standard, followed by Soxhlet extraction with acetone. The extract was concentrated to a few ml using a rotary evaporator set at a pressure of 39.9 kPa and a temperature of 40 °C, then a solvent was removed under the nitrogen flow gently, and a 0.5 ml of toluene was added to extract finally. One-fifth of the concentrated extract was measured and re-dissolved in 30 mL hexane and once again concentrated to ca. 3 ml with a rotary evaporator set at a pressure of 23.9 kPa and a temperature of 40 °C. Then, a 3 ml whole volume of the hexane solution was added to a preconditioned silica gel cartridge and eluted with 6 mL of dichloromethane-hexane (8:2 (*v*/v)). An aliquot (100 μL) of *n*-nonane was added to the eluent, and the resulting solution was then concentrated to 100 μL under nitrogen flow gently. Finally, the solution was transferred to an insert vial. PAHs were analyzed by GC/MS (Model 5973 N, Agilent Technology, California, USA) in the selected ion monitoring mode (injection port temperature 300 °C, injected volume 1 μL, and GC temperature profile as follows: initial temperature: 50 °C for 5 min; the temperature was raised by 15 °C/min until it reached 185 °C; from 185 °C, the temperature was raised by 8 °C/min until it reached 320 °C, at which it was maintained for 22 min. Ionization was in the EI mode. PAHs were determined by the internal standard method using PAHs deutriumed.

**Table 1:** Information on the PAHs measured in this study

PAHs	Abbreviation	Number of benzene rings	Vapor pressure* (Pa at 25 °C)	Target ion mass (m/z)	Retention time (min)	IARC classify	Mutagenicity**
Naphthalene	Nap	2	10.4	128	9.6	2B	–
Acenaphthylene	Acy	3	8.9 × 10^–1^	152	12.6	–	(?)
Acenaphthene	Ace	3	2.9 × 10^–1^	153	12.8	–	(?)
Fluorene	Flu	3	9.0 × 10^–2^	166	13.9	3	–
Phenanthrene	Phe	3	1.6 × 10^–2^	178	16.5	3	(?)
Anthracene	Ant	3	8.0 × 10^–4^	178	16.6	3	–
Fluoranthene	FLN	4	1.2 × 10^–3^	202	20.0	3	+
Pyrene	Pyr	4	6.0 × 10^–4^	202	20.8	3	(?)
Benzo [a]anthracene	BaA	4	2.8 × 10^–5^	228	24.5	2A	+
Chrysene	Chy	4	8.4 × 10^–5^(at 20 °C)	228	24.8	3	+
Benzo [b]fluoranthene	BbF	5	6.7 × 10^–5^	252	27.8	2B	+
Benzo [k]fluoranthene	BkF	5	1.3 × 10^–8^(at 20 °C)	252	27.9	2B	+
Benzo [a]pyrene	BaP	5	7.3 × 10^–7^	252	29.0	2A	+
Dibenzo [a,h]anthracene	dBahA	5	1.3 × 10^–8^	278	33.3	2A	+
Indeno[1,2,3-c,d]pyrene	IndP	6	1.3 × 10^–8^	276	33.3	2B	+
Benzo [ghi]perylene	BghiP	6	1.4 × 10^–8^	276	35.0	3	+

*: International programme on chemical safety environmental health criteria 202 selected non-heterocyclic policyclic aromatic hydrocarbons Table 4 [37]

**: International programme on chemical safety environmental health criteria 202 selected non-heterocyclic policyclic aromatic hydrocarbons Table 2 [37]

+: Positive, −: Negative, (?): Questionable

### Mutagenicity test by the microsuspension method

One-fourth of both quartz-fiber filters for PM2.5 and PM > 2.5 of HV method were used for bioassay by the microsuspension method. The organic fractions were extracted by ultrasonication (Ultra Sonic Cleaner AU-501CO; AIWA Medical Industrial Corporation, Tokyo, Japan) with acetone twice. The two extracts were combined and then concentrated to a few mL using a rotary evaporator. The solvent was removed under a nitrogen flow gently, and the dried samples were stored at − 80 °C until assayed.

After defrosting at room temperature [23], an extracted sample was dissolved in DMSO and tested for mutagenicity. The microsuspension method, using the sensitized Ames test, was used for the mutagenicity assay. It was carried out with S9 mix addition and non-addition conditions using *S. typhimurium* strains TA98 and TA100 following the protocols used in previous studies [15, 16, 31, 33]. When the dose-response relationship was obtained, the case of the colony by which the number of obtained return mutation colonies is beyond 2 times of the control value was defined as positiveness and 1.5-two times, as weak positiveness and less than 1.5 were difined as negative.

## Results and discussion

### Carbonaceous and inorganic chemicals in PM2.5

**Fig. 2:**
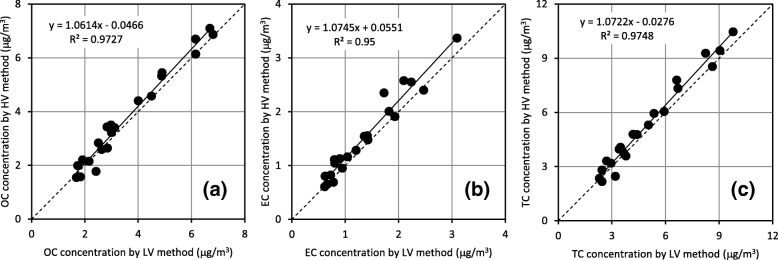
Comparison of (**a**) elemental carbon (EC), (**b**) organic carbon (OC), and (**c**) total carbon (TC) concentrations measured with HV and LV methods. The solid line presents the regression line by at least two multiplication. The broken line presents the line equivalent in HV method and LV method

**Fig. 3:**
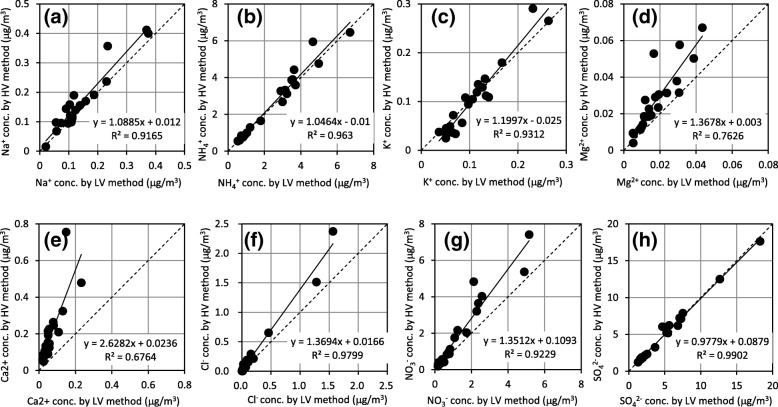
Comparison of (**a**) Na^+^, (**b**) NH_4_^+^, (**c**) K^+^, (**d**) Mg^2+^, (**e**) Ca^2+^, (**f**) Cl^−^, (**g**) NO_3_^−^, and (**h**) SO_4_^2−^ concentrations measured with HV and LV methods. The solid line presents the regression line by at least two multiplication. The broken line presents the line equivalent in HV method and LV method

The EC concentration was 1.4 ± 0.7 μg/m^3^, and the OC concentration was 3.7 ± 1.8 μg/m^3^ in PM2.5 collected by the HV method. The atmospheric EC and OC concentrations measured in this study were at a level similar to those in other reports of Japanese atmospheric EC and OC [9, 11, 12, 24]. The ion concentrations in PM2.5 collected by the HV method for Na^+^, NH_4_^+^, K^+^, Mg^2+^, Ca^2+^, Cl^−^, NO_3_^−^ and SO_4_^2−^ were 0.14 ± 0.10, 2.47 ± 1.88, 0.10 ± 0.07, 0.02 ± 0.02, 0.07 ± 0.16, 0.20 ± 0.58, 1.37 ± 2.04, and 4.87 ± 4.22 μg/m^3^, respectively. The atmospheric ion concentrations measured in this study were similar to those in other reports on Japanese atmospheric ion species [20, 30]. The results from the HV and LV methods were compared in Figs. 2 and 3. Figure 2 showed that there was a close agreement between concentrations of carbonaceous components in PM2.5 from the HV method and LV method. Furthermore, the slopes of the regression lines were in the range of 1.06 to 1.07, very close to 1. In Fig. 3, a close agreement was shown for Na^+^, NH_4_^+^, K^+^, and SO_4_^2−^ between the two methods, but the concentrations of Mg^2+^, Ca^2+^, Cl^−^, and NO_3_^−^ were not similar between the two methods. The concentrations of Mg^2+^, Ca^2+^, Cl^−^, and NO_3_^−^ by the HV method were about 1.4, 2.6, 1.4, and 1.4 times higher, respectively, than those by the LV method. Uchiyama reported that the ion concentrations in SPM differed depending on particle size, with the NH_4_^+^, SO_4_^2−^, and K^+^ concentrations in the fine particles being about 10, 4, and 2 times higher than those in coarse particles, in the other hand, Ca^2+^, Mg^2+^, and Na^+^ concentrations in coarse particles were about 10, 5, and 3 times higher than those in fine particles [34]. Because the impactor couldn’t be divided among the particle diameter perfectly for 50% cut-off value, it was suggested that a possibility with some properties with HV method tends to undergo influence of a particle large compared with LV method.

### Polycyclic aromatic hydrocarbons (PAHs) in PM2.5

The PAH concentration per cubic meter of total sampling flow, collected as particulate components using the LV and HV methods, was measured and compared. These results are shown in Table 2. The atmospheric PAH concentrations measured in this study are at a level similar to those in other reports on Japanese atmospheric PAHs [22, 38]. The comparison of the PAH concentrations of PM2.5 between the HV and LV methods was shown in Table 2 in which the results were classified by the vapor pressure of PAHs. The results demonstrated significant correlation with significant level < 0.01 (R = 0.795〜0.985) for low-vapor-pressure PAHs (less than 10^− 4^ Pa) between the concentrations associated with PM2.5 by the LV and HV methods. In contrast, for PAHs, the vapor pressure of which was greater than 10^− 4^ Pa, the concentrations obtained by the HV method were less than those obtained by the LV method. The linear velocity of filter surface by HV method is 28.7 cm/sec, is 1.23 times higher than that of LV method. A little difference was recognized between HV method and LV method. And, because of the shape of the suspended particulate has a distorted shape, not globular, the particles were separated by an aerodynamic diameter, could not separate correctly by particle size. Therefore, it is consider that the coarse particle near the PM2.5 influence PM2.5 fraction. The factor for the difference is not clear, so cause investigation is need urgently. But, HV method could collect low-vapor-pressure PAHs (less than 10^− 4^ Pa) selectively, This means that the HV method is suitable for low-vapor-pressure PAHs (less than 10^− 4^ Pa), which are mutagenic or carcinogenic [13].

**Table 2:** The atmospheric PAH concentration levels as determined by the LV and HV methods

Abbreviation of PAH	Vapor pressure (Pa at 25 °C)	Atmosphereic concentration by LV method ave. ± SD; ng/m^3^)	Atmosphereic concentration by HV method (ave. ± SD; ng/m^3^)	R value
Nap	10.4	1.76 ± 0.73	0.70 ± 0.42	−0.369
Acy	8.9 × 10^–1^	40.1 ± 20.9	0.47 ± 0.46	−0.058
Ace	2.9 × 10^–1^	0.24 ± 0.07	0.04 ± 0.03	−0.144
Flu	9.0 × 10^–2^	0.25 ± 0.17	0.05 ± 0.02	0.344
Phe	1.6 × 10^–2^	3.79 ± 0.81	0.45 ± 0.21	0.649
Ant	8.0 × 10^–4^	0.11 ± 0.08	0.03 ± 0.02	0.077
FLN	1.2 × 10^–3^	3.32 ± 0.85	0.49 ± 0.25	0.265
Pyr	6.0 × 10^–4^	1.71 ± 0.96	0.29 ± 0.15	0.068
BaA	2.8 × 10^–5^	0.16 ± 0.12	0.13 ± 0.12	0.795
Chy	8.4 × 10–5(at 20 °C)	0.54 ± 0.34	0.44 ± 0.35	0.906
BbF	6.7 × 10^–5^	0.31 ± 0.29	0.40 ± 0.29	0.951
BkF	1.3 × 10^–8^(at 20 °C)	0.17 ± 0.12	0.16 ± 0.13	0.928
BaP	7.3 × 10^–7^	0.25 ± 0.14	0.19 ± 0.15	0.954
dBahA	1.3 × 10^–8^	0.03 ± 0.02	0.03 ± 0.02	0.944
IndP	1.3 × 10^–8^	0.22 ± 0.19	0.25 ± 0.20	0.985
BghiP	1.4 × 10^–8^	0.33 ± 0.27	0.28 ± 0.22	0.964

R means the coefficient of correlation

### Highly carcinogenic PAHs in PM2.5

In this study, DBalP, which is highly carcinogenic and has six benzene rings, was also measured by GC/MS. DBalP is not included in the U.S. Environmental Protection Agency (U.S EPA) priority 16 PAHs; however, it is notable that the relative potency factor (RPF) [35] of DBalP is 30 [19]. DBalP could be detected in the PM2.5 samples by the HV method, but it was not detected in PM2.5 by the LV method. The atmospheric concentration of DBalP ranged from 0.014 to 0.078 ng/m^3^, and this concentration was about one-tenth that of BaP. DBalP concentrations in present study were one-tenth of lower than that in China (Wei [17]) by three days sampling, and were similar to those in Chiba prefecture [14] by one week sampling. The measurement of low concentration PAHs by HV method could be shortened sampling time to 24 h from a few days successfully. Then, the comparison of mutagenic potency of DBalP and BaP calculated by their concentration and PRF showed that the atmospheric carcinogenic potency of DBalP is three times higher than that of BaP. It is a recognized that the HV method is suitable for the quantification of one of the most-highly carcinogenic PAHs. The major advantage of the HV method is the extreme microanalysis of PM2.5 composition which is possible given the large amount of PM2.5 available from the HV method, a process which is impossible to detect with the small amount of PM2.5 collected by the LV method.

### Mutagenicity of PM2.5

A LV method was used for conventional PM2.5 collection and analysis of airborne particles, but the sample size achieved by this method was very small. Because nitro-PAHs were reported as mutagen which could be present in airborne particles, the *S. typhimurium* strain, YG1024, developed to exhibit very high sensitivity to nitro-PAHs, was used to assay for mutagenicity in such small airborne particle samples. YG1024 strain was reported to exhibit approximately 10 times greater sensitivity to mutagenesis from food and environmental samples than did the TA98 strain [10, 21]. But it was considered that mutagenic evaluation with YG1024 had an in-built TA1537 (or TA97 and TA97a), TA98, TA100 and WP2uvrA (or WP2uvrA (pKM101) or TA100) have been used for mutagenic assay (OECD 1997). The YG1024 strain is not selected for standard mutagenicity assays. So, in this study, two types of strain were used, one is TA98 strain that detected mutagenicity of the frame-shift type, another is TA100 that detected mutagenicity of the base substitution type. Furthermore, the measurements of the mutagenic activity in PM2.5 samples were tried here using the microsuspension method [15] that exhibits greater sensitivity than does the original Ames test.

In previous studies, the sample of mutagenic assay for environmental atmosphere using TA98 and TA100 used to be collected by HV method for 24 h. The sample of 1/3 to 1/2 was used for mutagenic assay, that equivalent to air volume 400 m^3^ to 600 m^3^ [5]. And, Matsumoto et al. carried out sampling for mutagenic assay for atmosphere by LV method for 3 days and collected air volume120 m^3^ to 240 m^3^ [18]. Because of these air volume were equivalent to sampling volume for 5 days to 10 days by LV method, it was impossible to elucidate diversification of a testing condition and fluctuation during the day.

In this study, the mutagenic evaluation for environmental atmosphere using TA98 and TA100 strain was attempt by combining microsuspention sensitized Ames test and large volume sampling method.

The air volume necessary to detection of the positive and weak positive of mutation are indicated in Table 3. In the case of actual assay, the necessary air volume was four times of value in Table 3, because mutation assay need assay with different 5 concentrations and duplicate measurement. Its air volume was different depending on samples by the percentage of the mutagenic substance included in a sample, 14 m^3^ (actually, 56 m^3^) was needed by 4 conditions by the detection rate of the 50% in present study. This air volume was equivalent to the sample for 48 h by LV method. Additionally, the air volume necessary to detection of 90% was approximately 140 m^3^, this volume was equal to the suction amount of the previous study.

**Table 3:** Comparison of the air volume (m^3^) necessary to false positive in present study (in the case of single dose)

					(unit: m^3^)
	TA100 - S9mix	TA100 + S9mix	TA98 – S9mix	TA98 + S9mix	Total
average	7.85	4.65	1.12	4.45	18.07
sd	5.98	4.07	1.31	4.40	12.72
50% of detection parcentage	6.36	3.40	0.99	3.06	14.09
90% of detection parcentage	14.95	7.96	1.74	12.57	35.59
95% of detection parcentage	18.70	12.54	2.05	14.15	50.22
All	32.28	26.59	10.67	17.88	59.19

**Fig. 4:**
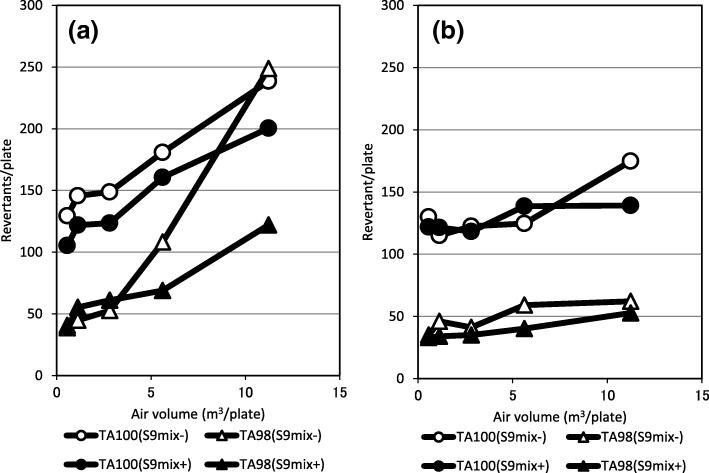
Typical dose–response curves of mutation assay for PM2.5 in Tsukuba in (**a**) 30th July, 2012 and (**b**) 8th August, 2012.a: Results of typical positive mutagenicity. b: Results of negative, false-positive and positive mutagenicity. TA100 S9mix- and TA98 S9mix + were negative, TA100 S9mix + was false-positive and TA98 S9mix- was positive

**Fig. 5:**
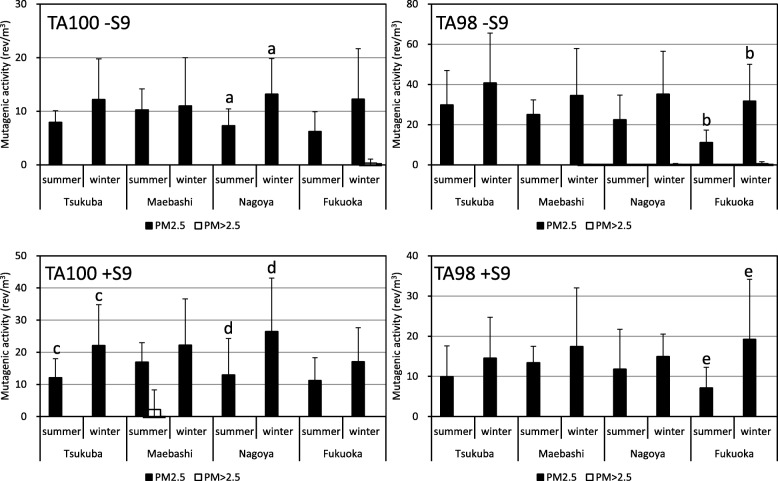
Comparison of mutation activity in PM2.5 and PM > 2.5 per unit air volume at different sites and seasons. *: Results with a common letter present significant differences (*p* < 0.05)

**Fig. 6:**
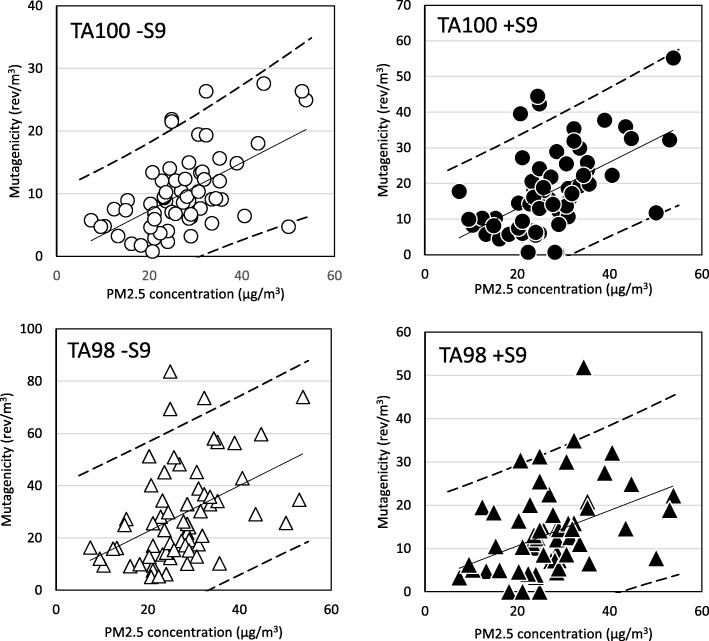
Relationship between mutagenicity and PM2.5 concentration The solid line presents the regression line, the both broken lines present the 95% prediction interval

Samples of PM2.5, taken at four locations from the Kanto area to the Kyushu area, were collected by the HV method. Then, the mutagenic activities of these PM2.5 samples were tested using the microsuspension method with TA98 and TA100 strains. Typical results of the mutagenicity assay are shown in Fig. 4. In Fig. 4 a, mutagenicity were positive in all four conditions. But, mutagenicity were negative in the conditions of TA100 without S9mix (TA100 S9mix-) and TA98 with S9mix (TA98 S9mix+), was weak positive in the condition of TA100 with S9mix (TA100 S9mix+) and was positive in the condition of TA98 without S9mix (TA98 S9mix-). An outline of the detection rate of mutagenic activity in PM2.5 is shown in Table 4. The positive rate, including weak positives, was 98.4% by TA100 (-S9mix), 95.3% by TA100 (+S9mix), 100% by TA98 (-S9mix) and 92.2% by TA98 (+S9mix). The average mutagenic activities per unit volume at each location are shown in Fig. 6. In spite of PM > 2.5 particles accounting for about 20% of the particle weight of the sample, mutagenic activity in PM > 2.5 was very weak (Fig. 6). It was considered that most of mutagenic compounds were associated with the fine particles like PM2.5 in the atmosphere (Fig. 5). Significant differences in mutagenic activity of PM2.5 between summer and winter were recognized in Nagoya and Fukuoka in spite of it being recognized that there were no significant differences in particle concentrations between summer and winter. The average (mean ± SD) concentration of PM2.5 was 24.0 ± 2.9 μg/m^3^ in summer and 24.6 ± 9.3 μg/m^3^ in winter at Tsukuba, 28.7 ± 2.8 μg/m^3^ in summer and 26.8 ± 13.8 μg/m^3^ in winter at Maebashi, 31.2 ± 5.1 μg/m^3^ in summer and 28.2 ± 13.9 μg/m^3^ in winter at Nagoya, and 25.8 ± 5.3 μg/m^3^ in summer and 18.2 ± 15.0 μg/m^3^ in winter at Fukuoka. Furthermore, the relationship between atmospheric PM2.5 concentration and mutagenicity is shown in Fig. 6. The correlation coefficients between PM2.5 concentration and mutagenic activity were significant (*p* < 0.05, *n* = 64) for each assay, namely 0.568 for TA100-S9mix, 0.525 of TA100 + S9mix, 0.445 of TA98-S9mix, and 0.391 of TA98 + S9mix. On the other hand, it was found that mutagenic activity differed significantly, in spite of the similar PM2.5 concentrations being reported from the different sites over the two seasons. From the relationship between PM2.5 concentration and mutagenicity, it is apparent that more data need to be collected and the distribution analyzed to identify management concentrations.

**Table 4:** Detection rates of mutagenic activity by the microsuspension method

	Tsukuba		Maebashi		Nagoya		Fukuoka	
	number	parcentage(%)	number	parcentage(%)	number	parcentage(%)	number	parcentage(%)
Negative	3	(4.7)	1	(1.6)	2	(3.1)	3	(4.7)
week positive	13	(20.3)	18	(28.1)	13	(20.3)	11	(17.2)
Positive	48	(75.0)	45	(70.3)	49	(76.6)	50	(78.1)
sum	64	(100.0)	64	(100.0)	64	(100.0)	64	(100.0)

The concentration of PM2.5 in the atmosphere is regulated globally because of the detrimental impact of these particles on human health. On the other hand, since the composition of the chemical substances associated with PM2.5 differs according to the sources, it can also be used in source analysis by the two commonly used receptor models, the chemical mass balance (CMB) model and the positive matrix factorization (PMF) model. In this study, the HV method could detect significant differences in mutagenic activity between summer and winter samples, although there was no significant difference between seasons in the concentration of PM2.5 at the same location. These results suggest that the components of particle-based pollutants related to carcinogenesis in PM2.5 could change depending on the season. Therefore, it is considered that the human health risk posed by PM2.5 would also vary, depending on the composition of the substances, but these effects have not yet been clarified in detail. The HV method of fine particle collection can evaluate the risk of PM2.5 precisely. Since the toxic effects of PM2.5 extend to cardiovascular and respiratory diseases, the measurement of chemical substances related to such diseases and the determination of the end-points of these compounds are urgently required. It is considered that the HV method proposed in this study is a very useful tool for the comprehensive evaluation of the health effects of carcinogens by the mutation assay and by precise measurement of trace chemicals in the fine particles.

## Conclusions

A strong harmful effect is attributed to a fine particle (PM2.5) in the atmosphere because PM2.5 has a high deposition rate in the respiratory organs. In present study, we would propose a new PM2.5 sampling method (HV method) using high-volume air sampler with an impactor and compared and evaluated it using the Low-volume air sampler based authentication method (LV method). The results showed that the HV method had the same performance with LV method in terms of measuring the inorganic components (OC, EC, Na^+^, NH_4_^+^, K^+^ and SO_4_^2−^) and low-volatile PAHs that are mutagenic or carcinogenic. Because of the air volume collected by HV method was forty times higher than the LV method, the high carcinogen and mutagen like DBalP that could be not detected by LV method were detected at 0.014〜0.078 ng/m^3^. Furthermore, HV method with microsuspension method could evaluate the mutagenic activity of PM2.5 by TA98 and TA100 strain. The HV method for PM2.5 proposed in present study is very useful for detecting targeted micropollutants present at very low concentrations in the air. On the other hand, for low molecular weight PAH and some inorganic ionic species (Mg^2+^, Ca^2+^, Cl^−^ and NO_3_^−^), the HV method tended to show higher concentration than the LV method. Attention should be paid according to the application.

## Abbreviations

BaP: Benzo [a]pyrene
CMB: The chemical mass balance
DBalP: Dibenzo [a,l]pyrene
DMSO: Dimethyl sulfoxide
EC: Elemental carbon
GC/MS: Gas chromatography–mass spectrometry
IMPROVE: The Interagency Monitoring of Protected Visual Environments
LV: Low-volume air sampler
OC: Organic carbon
PAHs: Polycyclic aromatic hydrocarbons
PM > 2.5: Particle bigger than PM2.5
PM2.5: Fine particles with an aerodynamic diameter less than 2.5 μm
PMF: The positive matrix factorization
RPF: Related potency factor
SPM: Suspended particulate matter
WHO: The World Health Organization

## References

[1] Błaszczyk E, Rogula-Kozłowska W, Klejnowski K, Fulara I, Mielzynska-Svach D. Polycyclic aromatic hydrocarbons bound to outdoor and indoor airborne particles (PM2.5) and their mutagenicity and carcinogenicity in Silesian kindergartens, Poland. Air Qual Atmos Health. 2017;10:389–400. 10.1007/s11869-016-0457-5 PMC5348569 28356998

[2] Chow JC, Watson JG, Crow D, Lowenthal DH, Merrifield T. Comparison of Improve and Niosh carbon measurements. Aerosol Sci Technol. 2001;34:23–34.

[3] Claudia RR, Sergio MC, Jose LM, Claudia AFA, Israel F (2013). Genotoxicity of Polycyclic Aromatic Hydrocarbons and Nitro-Derived in Respirable Airborne Particulate Matter Collected from Urban Areas of Rio de Janeiro (Brazil). BioMed Res Int.

[4] Dockery DW, Pope CA, Xu XP, Spengler JD, Ware JH, Fay ME, Ferris BG, Speizer FE (1993). An association between air-pollution and mortality in 6 United-States cities. N Engl J Med.

[5] Endo, O., Goto, S., Matsumoto, Y., Sakai, S., Akutagawa, T., Asanoma, M., Hirayama, T., Watanabe, T., Tsukatani, H., Sera, N., Tada, A. and Wakabayashi, K.: Mutagenicity of airborne particles, river waters and soils in Japan from 1996 to 2003. Environ Mutagen Res, Vol.26, pp9–22 (2004).

[6] Endo, O., Sugita, K., Goto, S., Amagai, T. and Matsushita H.: Mutagenicity of size-fractionated airborne particles collected with Andersen low pressure impactor, L Health Sci Vol.49, No.1, pp22–27 (2003).

[7] Ezoe, Y., Goto, S., Tanabe, K., Endo, O., Koyano, M., Watanabe, I. and Matsushita, H.: Polycyclic aromatic hydrocarbon concentrations of airborne particles in urban air over the past twenty years. Polycyclic Aromatic Comounds, Vol.24, pp635–646 (2004).

[8] Fu J, Jiang D, Lin G, Liu K, Wang Q (2017). An ecological analysis of PM2.5 concentrations and lung cancer mortality rates in China. BMJ Open.

[9] Fujikawa, K., Shigekazu, Y., Shiro, T., Hisao, C., Okihiro, O. and Shinji I.: The behavior of carbon compounds (EC,OC) in aerosols, and its relationships with other compounds. - analysis of daily data. Annual Reports of Fukuoka Institute of Health and Environmental Sciences, Vol.35, pp93–97 (2008).

[10] Gabbani G, Nardini B, Bordin A, Pavanello S, Janni L, Celotti L, Clonfero E (1998). Urinary mutagenicity on TA98 and YG1024 *Salmonella typhimurium* strains after a hamburger meal: influence of *GSTM1* and *NAT2* genotypes. Mutagenesis.

[11] Hashimoto, T.: Investigation of PM2.5 measurements in the environmental atmosphere of Kagawa prefecture (II). Annual Report of Kagawa Prefectural Research Institute for Environmental Sciences and Public Health, Vol.12, pp45–55 (2013).

[12] Hoshino, T., Kumagai, K., Yamaguchi, N. and Saito, Y.: Investigation of fine particulate pollution in atmosphere in Gunma. Annual Reports of Gunma Prefectural Institute of Public Health and Environmental Sciences, Vol.43, pp47–51 (2011).

[13] IARC (2010). IARC Monographs on the Evaluation of Carcinogenic Risks to Humans. Some Non-heterocyclic Polycyclic Aromatic Hydrocarbons and Some Related Exposures.

[14] Ichikawa Y, Watanabe T, Horimoto Y, Ishii K, Naito S (2018). Measurements of 50 non-polar organic compounds including polycyclic aromatic hydrocarbons, n-alkanes and phthalate esters in fine particulate matter (PM2.5) in an industrial area of Chiba prefecture, Japan. Asian J Atmos Environ.

[15] Kado NY, Guirguis GN, Flessel CP, Chan RC, Chang KI, Wesolowski JJ (1986). Mutagenicity of fine (less than 2.5 microns) airborne particles: diurnal variation in community air determined by a *Salmonella* micro preincubation (microsuspension) procedure. Environ Mutagen.

[16] Kado, N.Y., Langlay, D. and Eisenstadt, E.: A simple modifyion of the *Salmonella* liquid-incubation assay. Increased sensitivety for detecting mutagens in human urine. MutatRes, Vol.121, No.1, pp25–32 (1983). 10.1016/0165-7992(83)90082-9 6306458

[17] Li W, Wang C, Wang H, Che J, Shen H, Shen G, Huang Y, Wang R, Wang B, Zhang Y, Chen H, Chen Y, Su S, Lin N, Tang J, Li Q, Wang X, Liu J, Tao S (2014). Atmospheric polycyclic aromatic hydrocarbons in rural and urban areas of northern China. Environ Pollut.

[18] Matsumoto, O., Ando, M., Tamura, K.,: Differences of mutagenic activity od Airborn particulates by particle size –assay by the *Salmonella* Mirosuspention procedure-. Jpn J Toxicol Environ Healty Vol.39, No.2, pp139–147 (1993).

[19] Minnesota department of Health (2016). Guidance for Evaluating the Cancer Potency of Polycyclic Aromatic Hydrocarbon (PAH) Mixtures in Environmental Samples.

[20] Miyoshi T, Akiyama K, Ueno H, Yokota H, Kouichiro Ishi K, Ishi M, Ito Y, Higuchi Y. Research on PM2.5 in the atmosphere. Annual Reports of Tokyo Metropolitan Research Institute for Environmental Protection; 2009. p. 110–3.

[21] Nagai A, Kano Y, Funasaka R, Nakamuro K (2002). Mutagenic characteristics and contribution of polycyclic aromatic hydrocarbons to mutagenicity of concentrates from Municipal River water by blue chitin column. J Health Sci.

[22] Nozaki, K., Kushida, M., Motoki, S. and Suzuki, K.: Study on the polycyclic aromatic hydrocarbon compounds present in particulate matters in the atmosphere. Annual Report of Kagawa Prefectural Research Institute for Environmental Sciences and Public Health, Vol.6, pp45–51 (2007).

[23] OECD: OECD guideline for testing of chemicals Test No. 471: Bacterial reverse mutation test (1997).

[24] Oura, Y., Sugawara, S., and Ebihara, M.: Determination of elemental and organic carbon in atmospheric suspended particulate matters using photon activation analysis. Research Report of Laboratory of Nuclear Science, Vol.41, pp71–76 (2008).

[25] Peters, T.M., Vanderpool, R.W., and Wiener, R.W.: Design and calibration of the EPA PM2.5 well impactor Ninety-Six (WINS). Aerosol Sci Technol, Vol.34, pp389–397 (2001).

[26] Sameton JM, Dominici F, Curriero FC, Coursac I, Zeger SL (2000). Fine particulate air pollution and mortality in 20 us cities, 1987-1994. N Engl J Med.

[27] Schwartz J (1996). Air pollution and hospital admissions for respiratory disease. Epidemiology.

[28] Sugita, K., Goto, S., Endo, O., Nakajima, D., Yajima, H. and Ishii, T.: Particle size effects on the deposition ratios of airborne particles in the respiratory tract. J Health Sci, Vol.50, No.2, pp185–188 (2004).

[29] Sugiyama H, Saito T (2004). Seasonal Variation and Size Distribution of Polycyclic Aromatic Hydrocarbons in Ambient Air. Bulletin of Kanagawa Environmental Research Center.

[30] Suzuki, Y., Kenji Goto, K. And Misawa, T.: Chemical characteristic analysis of PM2.5 in the ambient air on Kawasaki City (2012). Annual Report of Kawasaki Environmental Research Institute, Vol.1, pp31–36 (2013).

[31] Takagi Y, Goto S, Nakajima D, Endo O, Koyano M, Kohzaki K, Matsushita H (2002). Mutagenicity of suspended particulate matter divided in three sizes lndoors. J Health Sci.

[32] Takagi Y, Sugita K, Muto M, Kato Y, Kohzaki K, Endo O, Goto S (2004). Measurement of Polynuclea aromatic hydrocarbons in Canaine lung after alkaline decomposition. J Vet Med Sci.

[33] Tamagawa K, Aihara Y, Takahashi Y, Seki T (1988). Seasonal Variations of Mutagenic Activities of Airborne Particulates –Influence of Asphalt Dust Produced by Studded Tires of Automobiles. J Japan Soc Air Pollt.

[34] Uchiyama, S.: Seasonal variation in size distributions for major ionic species in the atmospheric aerosol. J Japan Soc Air Pollut Vol.25, No.1, pp77–84 (1990).

[35] United States Environmental Protection Agency. Development of a Relative Potency Factor (RPF) Approach for Polycyclic Aromatic Hydrocarbon (PAH) Mixtures. Washington D.C; 2011.

[36] Watanabe T, Hasei T, Kokunai O, Coulibaly S, Nishimura S, Fukasawa M, Takahashi R, Mori Y, Fujita K, Yoshihara Y, Miyake Y, Kishi A, Matsui M, Ikemori F, Funasaka K, Toriba A, Hayakawa K, Arashidani K, Inaba Y, Sera N, Deguchi Y, Seiyama T, Yamaguchi T, Watanabe M, Honda N, Wakabayashi K, Totsuka Y (2014). Air Pollution with Particulate Matter and Mutagens: Relevance of Asian Dust to Mutagenicity of Airborne Particles in Japan. Genes and Environ.

[37] WHO: International Programme on Chemical Safety Environmental Health Criteria 202 Selected Non Heterocyclic Polycyclic Aromatic Hydrocarbons. (1988)

[38] WHO (2005). WHO Air Quality Guidelines for Particulate Matter, Ozone, Nitrogen Dioxide and Sulfur Dioxide.

[39] Yamada, E,, Matoba, D., and Fuse, Y.: Analysis of polycyclic aromatic hydrocarbons contained in atmospheric particulates in Kyoto. Bunseki Kagaku, Vol.62, pp275–283 (2013).

[40] Zhang YL, Cao F (2015). Fine particulate matter (PM2.5) in China at a city level. Scientific Reports.

